# Overuse of reliever inhalers and associated healthcare utilization of asthma patients

**DOI:** 10.1038/s41598-020-76280-2

**Published:** 2020-11-05

**Authors:** Ronit Hadad, Diana Likhtenshtein, Nimrod Maimon, Tzahit Simon-Tuval

**Affiliations:** 1 Department of Health Systems Management, Guilford Glazer Faculty of Business and Management and Faculty of Health Sciences, Ben-Gurion University of the Negev, P.O.B. 653, 84105 Beer-Sheva, Israel; 2 Clalit Health Services, Beer-Sheva, Israel; 3 Division of Internal Medicine, Pulmonology Institute, Soroka University Medical Center, Beer-Sheva, Israel

**Keywords:** Respiratory tract diseases, Health care economics

## Abstract

Global Initiative for Asthma 2019 guidelines recommend to avoid strengthening patients’ reliance on relievers since they increase exacerbation risk. Our aim was to examine the association between reliever inhalers overuse and all-cause healthcare utilization (HCU). A retrospective study among Clalit Health Services (CHS) adult enrollees (n = 977) for 2012–2017. Reliever inhalers overuse was defined as consistent prescription refills of ≥ 3 canisters annually. Adherence to controllers was calculated using the proportion of days covered. HCU included: hospitalizations, diagnostic and surgical procedures, medications, emergency room (ER) visits, and clinic visits. 27% of the study population (n = 264) consistently refilled ≥ 3 relievers prescriptions annually, and had higher adherence to controllers (0.38 vs. 0.24, p < 0.001). Their total 6-year HCU costs were not higher than that of others ($5,550 vs. $5,562, p = 0.107). Most HCU components [including hospitalization (p = 0.405) and ER visits (p = 0.884)] were comparable; however, medication costs were higher ($1734 vs. $1504, p < 0.001). A multivariable ordered-logit model revealed that frequent and regular use of relievers was not associated with higher HCU costs (OR = 0.82, 95% CI 0.62–1.09, p = 0.175). Higher adherence to maintenance and reliever therapy (OR = 2.18, 95% CI 1.44–3.28, p < 0.001), other controllers (OR = 3.30, 95% CI 2.11–5.16, p < 0.001), and nebulized SABAs and SAMAs (OR = 1.08, 95% CI 1.02–1.14, p = 0.007) was associated with higher costs. Overuse of reliever inhalers was prevalent and associated with higher adherence to controllers, yet not associated with higher all-cause HCU. This highlights the need to examine the sources of elevated usage in order to develop intervention strategies to optimize pharmaceutical therapy of asthma patients.

## Introduction

Controlling asthma and reducing exacerbations requires a balance between controller and reliever medications; however, there is a gap between real-world practice and evidence-based guidelines^1^. In addition, adherence rates to controllers are suboptimal, ranging from 15 to 54%^2^. Suboptimal adherence to inhaled therapy stems from treatment-related, patient-related and disease-related factors^3^.

The Global Initiative for Asthma (GINA) 2019 guidelines^4^ signal a paradigm shift with respect to use of relievers for asthma management and recommend avoiding traditional algorithms of care that strengthen patients’ reliance on the short-acting β_2_-agonist (SABA) since its regular and frequent use increases the risk of exacerbations. This paradigm shift triggered our interest in examining the association between elevated use of reliever inhalers and healthcare utilization (HCU).

Most of the studies that examined the association between adherence to asthma therapy and HCU have focused on adherence to controllers^5–14^. In most of these studies, improved adherence to controllers was associated with decreased hospitalizations and emergency room (ER) visits^5,7,9,12,13^. However, this trend was inconclusive^8,10^ or not observed^6,14^ in others. In addition, all of these studies analyzed data of short-term follow-up periods^5–14^, and most analyzed asthma-related HCU^5,7–14^. Half of the studies^6–8,10,12^ did include the use of SABAs in their analyses, but as an outcome variable; thus, they did not draw conclusions with regard to the association between elevated SABA use and HCU. To the best of our knowledge, the study of Piecoro et al.^15^ is the only one that examined whether overuse of inhaled SABA in the absence of concurrent inhaled corticosteroid use was associated with an increased risk of ER visits and hospitalizations. The study revealed that daily use of an inhaled SABA was associated with an increase in asthma-related hospitalizations when compared to patients who did not use inhaled SABA on a daily basis or received inhaled steroids on a continuous basis. However, this result was based on one-year follow-up; thus, it could not address the issue of long-term regular and frequent use of reliever inhalers and its corresponding effect on HCU.

The goal of this study was to examine the association between elevated and consistent use of reliever inhalers in six years of follow-up and the corresponding HCU. This information may highlight the actual long-term economic burden associated with inappropriate and excessive use of relievers. The Israeli single-payer setting enabled us to provide reliable and comprehensive measures of HCU data for a representative cohort of patients.

## Methods

### Study design and setting

A retrospective cohort study was conducted among enrollees of the Southern District of Clalit Health Services (CHS), the largest HMO in Israel. The study was conducted in accordance with the amended Declaration of Helsinki. The CHS Internal Review Board—Helsinki Committee for Community Medicine approved the study protocol and participants’ informed consent was waived by this committee since it was a secondary analysis of de-identified dataset (#0061-18-COM2).

### Study population

The inclusion criteria were enrollees: (1) of the Southern District of CHS, (2) aged 18–65 in 2012, (3) diagnosed with asthma (ICD-9 codes 493.xx) before 2012, (4) with active diagnosis of asthma during the entire follow-up period, and (5) who refilled an average of ≥ 1 prescription of inhaled relievers (SABAs and short-acting muscarinic antagonists (SAMAs)) annually during the follow-up period. Exclusion criteria were: (1) concomitant diagnosis of at least one of the following diseases: AIDS, thalassemia, hemophilia, Gaucher disease, COPD, malignancy, or (2) consumption of Omalizumab injections (that implies severe asthma).

### Medication adherence estimation

All study variables were obtained from the computerized database of CHS. We estimated adherence to relievers and controllers separately. Adherence to relievers (100–200 puff of SABA and SAMA inhalers) was calculated as the number of canisters used. This measure was calculated for each of the follow-up years (2012–2017) and as an average for the entire period. As stated in the 2019 GINA guidelines^4^, consumption of ≥ three 200-puff canisters of SABA annually increases the risk of severe exacerbation. Thus, regular and frequent use of reliever inhalers was defined as consistent prescription refills of ≥ 3 canisters in each of the follow-up years. In addition, we calculated the annual average number of packages of nebulized SABAs and SAMAs. Adherence to controllers was calculated using the proportion of days covered (PDC) for each of the follow-up years and as an annual average for the entire period. The PDC was calculated by dividing the number of months that canisters/tablets were available to the patient (numerator) by 12 months (denominator). The PDC was calculated as the annual average PDC for maintenance and reliever therapy (MART), and as the annual average of the maximum PDC level of the following controllers: (a) inhaled corticosteroid (ICS) inhalers, (b) leukotriene receptor antagonists (LTRA), (c) ICS + long-acting β2-agonists (LABA) combinations excluding MARTs, and (d) Theophylline. This analysis excluded prescription refills of controllers’ therapy administered by nebulizers since this was negligible in our cohort and since estimation of PDC is not applicable for nebulized therapy.

### Estimation of healthcare utilization

CHS enrollees, like all Israeli citizens, are covered by generous and quite uniform universal health insurance that provides access to various healthcare services with no or relatively low co-payments. HCU cost estimates analyzed in our study included: hospitalizations, diagnostic procedures, medications, surgical procedures, ER visits, and outpatient clinic visits. Total cost was calculated as the sum of all these estimates. Cost estimates were adjusted to June 2018 prices and converted to US dollars (USD) using the June 2018 exchange rate of 3.65 Israeli shekels per 1 USD. The quantity of primary care physician visits (by type of visit, e.g. face-to-face or telephone-based) was estimated as well, yet not included in the total HCU costs. HCU data were calculated for the entire follow-up period (2012–2017).

### Data analyses

Data were analyzed using Stata software (version 15.1, Stata Corp, College Station, TX, USA). The Mann–Whitney U test was used to determine between-group differences in HCU and adherence to controllers and nebulized SABAs and SAMAs. A multivariable ordered-logit model was specified to analyze predictors of HCU costs. The core independent variable was consumption of reliever inhalers (annual prescription refills of ≥ 3 canisters vs. others). This association was analyzed in the presence of: adherence to MART, adherence to other controllers (excluding MART), and consumption of nebulized SABAs and SAMAs. In addition, the model was adjusted for potential confounders including: age, gender, ownership of voluntary supplementary health insurance, disease duration, the Charlson Comorbidity Index (CCI), and comorbidities [e.g. hypertension, and ischemic heart disease (IHD)]. P-values of < 0.05 determined statistical significance in all analyses.

## Results

Our cohort consisted of 977 asthma patients (Table 1). Of those, 264 (27%) consistently refilled ≥ 3 prescriptions of canisters of reliever inhalers during six years of follow-up. Table 2 presents the adherence to controllers and nebulized SABAs and SAMAs, by study group. Patients who consistently refilled ≥ 3 prescriptions of canisters of reliever inhalers annually did not have higher adherence to MART (0.27 vs. 0.15, p = 0.052), yet, surprisingly, they had higher adherence to other controllers (0.38 vs. 0.24, p < 0.001).

**Table 1:** Characteristics of study population.

Variable	Refilled ≥ 3 prescriptions of canisters of relievers annually	Refilled < 3 prescriptions of canisters of relievers annually	*P* value
n (%)	264 (27%)	713 (73%)	
% Male	45.1	37.9	0.041^a^
Age^b^	46.2 ± 9.3 (47, 26–61)	44.1 ± 9.8 (44, 23–61)	0.003^c^
Disease duration^b^	15.1 ± 3.5 (14, 6–41)	14.7 ± 4.6 (14, 6–46)	0.014^c^
Hypertension (%)	25.0	19.6	0.068^a^
Diabetes (%)	19.3	20.1	0.797^a^
IHD (%)	4.9	3.5	0.309^a^
Obesity (%)	40.9	36.5	0.203^a^
Kidney disease (%)	7.6	5.2	0.158^a^
Liver disease (%)	2.7	2.5	0.911^a^
CVA (%)	1.5	2.0	0.644^a^
Charlson comorbidity index ^b^	2.3 ± 1.2 (2, 1–6)	2.1 ± 1.2 (2, 0–9)	0.034^c^
Supplementary health insurance coverage (%)	83.0	73.6	0.002^a^

IHD, ischemic heart disease; CVA, cerebrovascular accident.

^a^Chi-square test.

^b^Values are mean ± SD (median, min–max).

^c^Mann-Whitney U test.

**Table 2:** Adherence to controller and nebulized SAMAs and SABAs, by study group.

	Refilled ≥ 3 prescriptions of canisters of relievers annually	Refilled < 3 prescriptions of canisters of relievers annually	*P* value
Annual average PDC of MART^a^	0.27 ± 0.36 (0.01, 0.54)	0.15 ± 0.26 (0.03, 0.17)	0.052
Max annual average PDC of controllers (excluding MART)^b^	0.38 ± 0.34 (0.34, 0.58)	0.24 ± 0.26 (0.13, 0.36)	< 0.001
Annual average number of packages of nebulized SAMAs and SABAs^c^	1.80 ± 8.34 (0.17, 0.75)	0.67 ± 2.19 (0.00, 0.50)	0.068

Values are mean ± SD (median, IQR).

^a^MART, maintenance and reliever therapy.

^b^These controllers include inhaled corticosteroids (ICS) inhalers, leukotriene receptor antagonists (LTRA), ICS + long-acting β2 -agonist (LABA) combinations inhalers, and Theophylline, and exclude controller therapy administered by nebulizers.

^c^Nebulized short acting beta_2_ agonist (SABA) and short-acting muscarinic antagonists (SAMA).

HCU of the study groups during 2012–2017 is presented in Table 3. The total HCU costs of patients who consistently refilled ≥ 3 prescriptions of canisters of reliever inhalers annually were not higher than that of other patients ($5550 vs. $5,562, p = 0.107). Unexpectedly, the hospitalization costs of these patients were not a core component of their HCU, and these costs were not higher than the hospitalization costs of patients who consumed less than three canisters of reliever inhalers annually ($741 vs. $944, p = 0.405). Similarly, no significant difference in the number of ER visits was observed between groups ($361 vs. $363, p = 0.884). Most of the other HCU components were comparable as well, including the two core components of HCU, namely, surgical procedure costs (p = 0.505), and diagnostic procedure costs (p = 0.387). However, medication costs were higher among this group compared to others ($1734 vs. $1504, p < 0.001; Table 3). As expected, this difference stemmed predominantly from higher consumption of medications for the respiratory system ($837 vs. $439, p < 0.001). Consumption of medications for the alimentary tract and metabolism ($274 vs. $191, p = 0.340), and nervous system ($130 vs. $149, p = 0.190) were relatively high in this group, yet not different from the utilization of the other patients. Conversely, consumption of general anti-infectives for systemic use ($119 vs. $205, p = 0.030) and antineoplastic and immunomodulating agents ($94 vs. $234, p = 0.044) was lower in patients with consistently elevated usage of reliever inhalers compared to others.

**Table 3:** Healthcare utilization of study population, by study group.

Variable	Refilled ≥ 3 prescriptions of canisters of relievers annually	Refilled < 3 prescriptions of canisters of relievers annually	*P* value^a^
Total healthcare utilization costs	5550 ± 6049 (3325,5140)	5562 ± 9229 (2944,4699)	0.107
Medication costs	1734 ± 2493 (1123,1077)	1504 ± 5655 (661,843)	< 0.001
Number of Rx	251 ± 170 (200,193)	164 ± 142 (112,140)	< 0.001
Surgical procedure costs	1166 ± 3383 (0,0)	980 ± 3339 (0,0)	0.505
Number of procedures	0.2 ± 0.5 (0,0)	0.2 ± 0.4 (0,0)	0.554
Diagnostic procedure costs	897 ± 1019 (538,1024)	956 ± 1101 (599,925)	0.387
Number of procedures	14.5 ± 13.0 (12,16)	15.8 ± 14.4 (12,16)	0.310
Hospitalization costs	741 ± 1815 (0,492)	944 ± 2863 (0,594)	0.405
Number of admissions	0.4 ± 0.9 (0,1)	0.6 ± 1.3 (0,1)	0.259
Number of days	1.2 ± 3.0 (0,1)	1.5 ± 4.6 (0,1)	0.419
Outpatients specialists’ consultation costs	593 ± 621 (435,607)	721 ± 776 (503,708)	0.030
Number of visits	21.3 ± 21.9 (15,24)	25.8 ± 26.2 (18,27)	0.019
Emergency room costs	361 ± 480 (196,475)	363 ± 488 (196,505)	0.884
Number of visits	2.0 ± 2.5 (1,3)	2.1 ± 2.6 (1,3)	0.746
One-day outpatient care costs	58 ± 281 (0,0)	94 ± 515 (0,0)	0.117
Number of visits	0.1 ± 0.4 (0,0)	0.2 ± 1.6 (0,0)	0.100
Number of primary care visits	126 ± 76 (103,82)	121 ± 80 (98,86)	0.219

Values are mean ± SD (median, IQR). Costs are in US Dollars.

^a^Mann-Whitney U Test.

The multivariable model (Table 4) shows that consistent prescription refills of ≥ 3 canisters of reliever inhalers annually was not associated with increased HCU costs (OR = 0.82, 95% CI 0.62–1.09, p = 0.175). In addition, this analysis revealed that higher adherence to MART (OR = 2.18, 95% CI 1.44–3.28, p < 0.001), other controllers (OR = 3.30, 95% CI 2.11–5.16, p < 0.001), and nebulized SABAs and SAMAs (OR = 1.08, 95% CI 1.02–1.14, p = 0.007) were associated with higher HCU costs.

**Table 4:** Multivariable ordered-logit model of determinants of total healthcare utilization costs.

Variable	OR	95% CI	*P* value
Refilled ≥ 3 prescriptions of canisters of reliever inhalers^a^ annually (vs. others)	0.82	0.62–1.09	0.175
Max annual average PDC of controllers (excluding MART)^b^	3.30	2.11–5.16	< 0.001
Annual average PDC of MART	2.18	1.44–3.28	< 0.001
Annual average consumption of nebulized SABAs and SAMAs	1.08	1.02–1.14	0.007
Age (+ 1 year)	0.96	0.94–0.98	< 0.001
Gender (male vs. female)	0.48	0.38–0.62	< 0.001
Ownership of voluntary supplementary health insurance	1.93	1.46–2.56	< 0.001
Charlson comorbidity index	1.61	1.37–1.91	< 0.001
**Comorbidities**			
Hypertension	1.91	1.37–2.68	< 0.001
IHD	4.80	2.24–10.32	< 0.001
Obesity	1.34	1.04–1.73	0.026
n	977		
Pseudo R^2^	0.087		

PDC, proportion of days covered; MART, maintenance and reliever therapy; IHD, ischemic heart disease; SABA, short acting beta_2_ agonist; SAMA, short-acting muscarinic antagonists.

^a^Reliever inhalers include inhaled SABAs and SAMAs, and exclude nebulized SABAs and SAMAs.

^b^Controllers included inhaled corticosteroids (ICS) inhalers, leukotriene receptor antagonists (LTRA), ICS + long-acting β2 -agonist (LABA) combinations inhalers, and Theophylline, and exclude controller therapy administered by nebulizers.

## Discussion

This study revealed that, counter to our expectations, regular and frequent use of reliever inhalers was not found to be an independent determinant of increased HCU. However, this inappropriate use of reliever inhalers was associated with higher adherence to controllers (excluding MART), and this higher adherence to controllers, in turn, was indeed associated with increased HCU. These results imply that regular and frequent use of reliever inhalers, observed in our study among more than one fourth of the study population, may be a proxy of a more comprehensive phenomenon of inappropriate use of pharmaceutical therapy for asthma management that may indicate uncontrolled asthma, which is associated with elevated HCU costs.

Elevated use of relievers indicates poor asthma control and may worsen the disease symptoms due to the fact that the immediate symptomatic relief may delay or even prevent patients from seeking medical help^16^. In a qualitative study that examined guideline implementation from the patients’ perspective, one interviewee described his tremendous dependence on the reliever therapy by saying that when he noticed he did not have the reliever inhaler with him, he immediately had an asthma attack^17^. Asthma patients learn quickly that SABA and SAMA use is the fastest way to get symptom relief, and reliever treatment becomes their main way to manage attacks^18^. In the last decade in Israel, patients have been able to submit an online request for a prescription (instead of scheduling face-to-face appointment with their primary physician). This innovative approach may have led to excessive use of prescription drugs in general and reliever inhalers specifically^19^. It may be possible to curb overuse of relievers if prescriptions for reliever inhalers can be obtained only through face-to-face visits with the primary care physician, who can estimate the actual need for the medication and ensure correct controller use.

Reliever overuse is expected to be associated with poor adherence to controllers, but there is inconsistent evidence in this regard. While there is evidence that improved adherence was associated with lower use of SABA^7^, there is also evidence of mixed results (depending on the controller therapy)^8,10^, or similar to our findings, evidence of a positive association between adherence to controllers and use of relievers^6^. An additional study that focused on estimating the overuse of MART^20^ revealed that, despite the clear guidelines, 10% of MART users were concurrently using SABAs. Incorrect use of controllers may underlie these findings. While intake of chronic medications administered as tablets is relatively easy, inhaler use is more complex (requiring synchronized breathing), and necessitates guidance and tailoring to each patient’s needs and abilities in order to minimize inhaler use errors^21^. A systematic review on errors in inhaler use found that the inhaler technique has not improved in the past 40 years^22^, and that frequent critical errors in inhaler use are still prevalent^22,23^.

Asthma guidelines recommend and encourage asthma patients to be educated for self-management by a personalized asthma action plan^21^, although in practice only 27% of adult asthma patients receive such plans^24^. Even so, when an action plan already exists, it is usually not patient-centered, and there is a gap between healthcare provider recommendations and actual use which, in turn, leads to uncontrolled disease and SABA overuse^25^. A systematic review of the cost-effectiveness of intervention strategies targeted at asthma patients found that patient education and self-management intervention were the most cost-effective measures^26^; thus, it may be that the combination of the two together with patient-centered care would result in minimal use of relievers, higher adherence to controllers, lower unnecessary HCU costs, and higher quality of life. Pharmacists are the last medical professionals that the patient encounters before he/she uses the inhaler. Community pharmacists who are part of a multidisciplinary team that design continuous educational workshops may increase adherence to controllers, minimize incorrect use of inhalers, and encourage self-management of asthma patients^27,28^.

Together with reliever overuse, suboptimal adherence to controllers and associated HCU is a worldwide concern, and extant research on this topic has not demonstrated unequivocal results^5–14^. The current study provides a different angle of the analysis of elevated HCU determinants among asthma patients by analyzing the independent role of regular and frequent use of reliever inhalers. We revealed that this use was not associated with increased HCU, including hospitalizations and ER visits. Reliever inhalers are generally administered through metered dose inhalers, which require patients to synchronize between puffing and inhaling. This may result in many wasted puffs due to retrying to use these inhalers effectively^23^. In addition, relievers prescription refills may not solely indicate overuse, but rather stem from regular replacement due to expiration dates or for ease of having multiple inhalers in frequented places (e.g. one reliever at home, one in the gym locker and one on the work desk). Finally, asthma patients may take relievers prophylactically before exercise or exposure to triggers. These three reasons may imply that overconsumption of inhalers may not necessarily indicate an elevated intake of the medication and poor asthma control, and thus may not be reflected in elevated hospitalization and ER visits. Further in-depth exploration of the sources of excess refills of reliever inhalers is still warranted.

Our results reveal that the most dominant component of HCU costs was medication costs, similar to other studies that examined asthma-related HCU costs^9,13,14^. However, we provide a description of all-cause HCU; thus, all pharmacological groups of pharmaceutical therapy were included. As hypothesized, medication costs were higher among those who consistently refilled ≥ 3 prescriptions of canisters annually compared to others, and mostly due to significantly higher utilization of medication for the respiratory system, which corresponds with the positive association with controllers and may indicate these patients’ inability to manage their disease.

Our analysis has several limitations. First, a prescription refill does not necessarily mean medication uptake. With asthma, this limitation may be even more salient since only appropriate use of the inhalers (purchased) assures their effectiveness. Second, our analysis relied on financial data that lack clinical information; thus, we could not adjust the multivariable analysis to asthma severity. We acknowledge that asthma-related clinical characteristics might contribute to variability in the HCU estimates. Third, cost estimates of HCU may not be generalizable to other healthcare systems, as practice patterns and tariffs may differ. Even so, this limitation does not weaken our analysis since our objective was to compare patterns of HCU of two groups of patients, rather than to provide the absolute cost estimates of this population.

Notwithstanding these limitations, our study contributes to the extant knowledge by examining the effect of consistent and elevated usage of reliever inhalers on HCU, which now may be of great interest following the paradigm shift in asthma pharmaceutical care suggested by the updated GINA guidelines. Our comprehensive analysis of all-cause HCU patterns of asthma patients during a long follow-up period of six years may highlight the actual economic burden associated with inappropriate and excessive use of asthma therapy. Our results emphasize the need for further study to examine the sources of elevated usage of relievers and the low adherence to controllers, as well as incorrect inhaler use. This may enable development of intervention strategies that will optimize pharmaceutical therapy of asthma patients.

## Data Availability

The data that support the findings of this study contains potentially identifiable patients’ information. Thus, following the requirements of CHS Ethics Committee, the data are not publicly available. Data requests may be sent to the corresponding author [TST].

## Abbreviations

CCI: Charlson Comorbidity Index
CHS: Clalit Health Services
CVA: Cerebrovascular accident
ER: Emergency room
GINA: Global Initiative for Asthma
HCU: Healthcare utilization
ICS: Inhaled corticosteroid
IHD: Ischemic heart disease
LABA: Long-acting β2-agonist
LTRA: Leukotriene receptor antagonists
MART: Maintenance and reliever therapy
PDC: Proportion of days covered
SABA: Short acting beta_2_ agonist
SAMA: Short-acting muscarinic antagonists
USD: US dollars
